# Material Extrusion to Manufacture Carbide-Based Advanced Cutting Tools

**DOI:** 10.3390/ma16216902

**Published:** 2023-10-27

**Authors:** Gonçalo Oliveira, Ana Senos, Cristina Fernandes, Daniel Figueiredo, Teresa Vieira

**Affiliations:** 1 Centre of Mechanical Engineering, Materials and Processes (CEMMPRE), University of Coimbra, Rua Luis Reis dos Santos 290, 3030-788 Coimbra, Portugal; teresa.vieira@dem.uc.pt; 2 Department of Materials and Ceramics Engineering CICECO—Aveiro Materials Institute, University of Aveiro, Rua Santiago, 3810-193 Aveiro, Portugal; anamor@ua.pt; 3 Palbit S.A., P.O. Box 4, 3854-908 Branca, Portugal; cfernandes@palbit.pt (C.F.); dfigueiredo@palbit.pt (D.F.)

**Keywords:** material extrusion, tungsten carbide, cermet, additive manufacturing, cutting tools, cooling

## Abstract

Material extrusion (MEX) allows for the production of advanced cutting tools with new internal cooling systems, which are suitable for new machining equipment. To produce cutting tools via this process, hardmetal and cermet feedstock must be prepared for the extrusion of 3D printing filaments. After shaping the 3D object (green), debinding and sintering must be performed to achieve densification. Defects and microstructural heterogeneities were studied according to the powder material. The present study shows that, although MEX is a viable solution for hardmetals, it needs to produce homogeneous filaments for cermets. The WC-Co bulk microstructures versus hardness were similar to the ones that were measured with pressing and sintering. While cermet (Ti(CN)/WC-Ni/Co) microstructures were heterogeneous, their hardness, when compared with that from the pressing and sintering manufacturing process, decreased significantly.

## 1. Introduction

With the market for cutting tools and additive manufacturing (AM) growing [1,2,3], the combination of both of these has many advantages. For the last ten years, there has been an increase in the research on AM with respect to tungsten carbide and cermets [4,5,6]. They have been tested with powder bed fusion (PBF) [7,8,9,10,11,12,13,14], binder jetting (BJT) [15,16,17], material extrusion (MEX) [18,19,20,21,22,23,24], and vat photopolymerization (VPP) [25]. Although they still underperform compared to the pressing and sintering method [26,27], they still possess advantages related to design freedom, which can justify the use of AM for certain applications. For example, by reducing the weight of a tool, one can help increase the cutting speed while also reducing the material costs of creating the cutting tool. At the same time, generative design can be used to optimize size vs. function. New cooling systems can be devised based on efficient flow theories, like the constructal and conformal systems [28,29,30]. These geometric changes can increase cutting tool life by keeping the cutting tip of the tool at a desired temperature, thus avoiding thermal degradation, wear, fracture, tolerance deviation, and chip clogging [31,32,33,34,35,36]. Moreover, in indirect additive manufacturing, infill patterns may reduce sintering defects by creating internal “chimneys” to help eliminate the organic binder during debinding in MEX technology.

Concerning the AM of hardmetals and cermets, there is a clear distinction in quality between the processes that simultaneously shape and consolidate, like PBF, and those that separate these production steps—such as BJT and MEX [4,5,6,11]. Selective laser sintering (SLS) is a PBF technology that uses a laser to selectively sinter powder while it is deposited layer by layer. In this process, heating and cooling rates are extremely fast [7,12], and particularly high energy outputs are needed to achieve sintering temperatures. In hardmetals and cermets, the high temperatures, which are reached in the center of the energy beam, are well above the melting temperature of cobalt; this entails evaporation during the consolidation process, which results in the embrittlement of the material [7,8,10,12,13,14]. The heating/cooling thermal cycles are so fast that the thermal shock induced is enough to result in the development of cracks and delamination [9]. The carbon equilibrium is also neglected in this process, and many deleterious phases are formed (e.g., η phase) [8]. Moreover, the particle shape should be spherical, with a narrow size distribution close to 10 µm, to ensure a uniform layer deposition. This is particularly challenging to achieve for hardmetal and cermet powder.

Currently, the AM technologies that show the best results for WC-Co and cermets, are BJT and MEX [15,16,18,19,21,37]. Both use organic binders to assist in the shaping step, and both are removed afterward by debinding. BJT requires a spherical hardmetal powder with high percentages of metallic binder (12 to 20 wt.% for WC-Co [15,16]). The 3D objects made by this process demonstrate microporosity and a low sintered density, but they also have the lowest geometrical tolerances [10,11]. On the other hand, MEX can be adapted to the following different feedstock deposition methods: filament, pellets, rod, or suspension [38]. Suspension MEX allows for bigger solid loadings and sintered densities, but it has difficulty with overhang structures since it is a viscous paste [21,39]. The filament method is more versatile, but it induces a bigger macroscopic porosity due to the printing strategy used [18,19,23,37].

In 2018, Berger et al. produced 3D objects from WC-Co powder with different particle sizes [19]. They used a fine particle size of 8.5 wt.% of Co and a medium particle size of 12 wt.% of Co. The binder only references the use of thermoplastic elastomers (TPE) with different molecular weights and a solid content of only 38 to 45 vol.%. When debinding under 600 °C, there would be an incomplete binder removal in their feedstock composition. “Free carbon” was present on the sintered parts, but these registered magnetic saturation values that were too high. The successfully debound parts showed a microstructure like the conventional one [40]; however, the absence of certain grain growth inhibitors (i.e., VC or Cr3C2) enabled the grain to grow more than was expected.

Lengauer et al. (2018) used cermet powder (Ti(C,N)-Co/Ni-based) and WC + 10 (wt.%) Co powder to produce filaments for MEX [18]. The authors mixed each main powder (50 vol.%) with a TPE as the master binder, and polypropylene (PP) functionalized with maleic anhydride as the backbone. The filaments were homogeneous, and the printed 3D objects possessed defects that were only related to the printing strategy. After debinding (solvent and thermal) and sintering, the 3D objects based on cermet and hardmetal had a volume reduction of around 22% and a microstructure like that of the conventionally fabricated ones [40]. In 2019, Lengauer et al. produced filaments from a ready-to-press (RTP) powder of cermet and WC-Co but with an unspecified feedstock polymeric binder. Their objective was to study the sintering process [17]. They noticed that using either H_2_ or N_2_ as the thermal debinding atmosphere had practically an identical effect, and no reaction was detected with the cermet powder. However, a certain degree of carbon residue was present when the N_2_ was in the environmental atmosphere. They noticed that, in the case of the cermets, there was a substantial difference in the magnetic saturation (MS) values. This could be explained by the trapped carbon from the residue, which led to the formation of an FCC phase and low solubility in the Co/Ni binder phase, hence increasing the MS values.

In 2019, Cerejo et al. made filaments with solid loadings of up to 48.5 vol.% [37]. They also used TPE as an additive component, but only in small percentages when compared with other authors. The main constituent was a commercial-grade binder with a mix of waxes and polyolefin-modified polyoxymethylene, and they used a small quantity of plasticizer to promote particle dispersion and filament flexibility. The feedstock was prepared at 180 °C with a 30 rpm blade speed and a mixing time of 30 min. A critical powder volume concentration (CPVC) test was performed to find the best solid loadings percentage for that binder; in addition, it was also found that an additive formula at 48.5 vol.% was the ideal value. The filament had a homogeneous distribution of powder particles and polymer, with only small aggregates of tungsten carbide and cobalt. A TGA analysis showed a gradual decomposition of the polymer, with a complete removal achieved below 600 °C. Printing optimization was fundamental in decreasing the number of printing defects, and the overall shrinkage was 26%.

More recently, in 2022, A. Bose et al. researched WC-Co 3D objects that were constructed using a cartridge 3D printer [23]. They used solvent and thermal debinding, with a feedstock that had low solid loadings of 45%. The shrinkage values were different in the Z direction, measuring 25%, while in the X and Y directions, they were 23%. The η phase was found in the microstructure.

**Figure 1:**
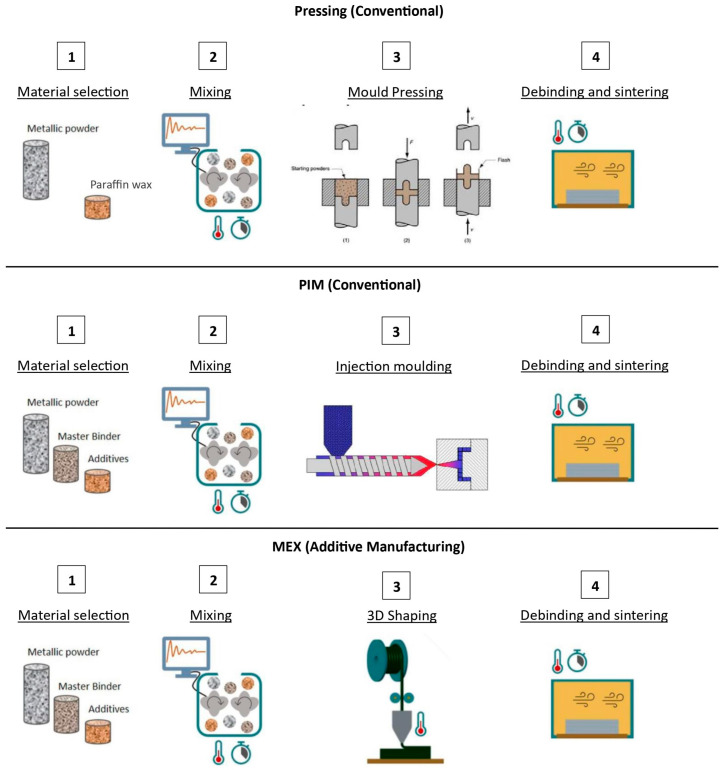
The material extrusion (MEX) process when compared with the conventional pressing and sintering method and the powder injection molding (PIM) (adapted) technique.

The design constraints of conventional processing, like the pressing and sintering method and PIM, can be overcome by AM processes like MEX (Figure 1). The internal design of tools can open new markets for tungsten carbide and metal (typically cobalt) if they can improve the current cooling solutions. Another big advantage is the ability to produce 3D objects that do not need to be fully densified, thus enabling lighter solutions with less powder use, which also translates into resource savings and more sustainable solutions. For the same number of components and similar geometry, which is possible with PIM or MEX, the shaping step will be more expensive for the first one (e.g., due to high equipment amortization). In the present study, the know-how necessary to make filaments, which are based on tungsten carbide and metal, is an added value due to high versatility being associated with a lower added cost. Nevertheless, the current limitations of MEX are surface roughness and finishing quality. In the conventional pressing and sintering method, production porosity is normally below 2% [26]. In PIM, porosity usually reaches between 2 to 5% without HIP, and below 2% with HIP [41,42]. With MEX, it is already possible to reach a 99.5% density when using a WC-Co paste [43].

Tungsten carbide (WC-Co) and MEX have been revealed as efficient binomials to use in response to the actual challenges of machining. However, the research work on cermets and MEX for similar applications (and where an efficient cooling system was necessary) included a deep study on MEX, in which the mixing of these different powder particles (TiCN/WC-Co/Ni/NbC/Mo_2_C) was achieved using WC-Co as the paradigm. The main objective of the present study is to highlight the role of different powder particles with dissimilar properties and characteristics, like the cermet composition, in the efficiency of MEX processing.

## 2. Materials and Methods

### 2.1. Powder, Binder, and Additives

The hardmetal powder (WC-Co) and a cermet (Ti(CN)/WC-Ni/Co) were provided by Palbit S.A. (Branca, Portugal). Their characteristics are summarized in Table 1.

Polymers, such as the poly(propylene-ethylene) copolymer (PEC) and polyamide (PA) (for the organic binder of the feedstock), were selected according to the properties required for the filament, including their processability. Other organic materials, such as paraffin wax (PW) and stearic acid (SA), were mixed as per the proportions shown in Table 2. PW is a very common binder for carbides due to its wettability, spread, low viscosity, processing temperature, and low carbon residue after debinding [41,44,45]. While it is sufficient for the pressing and sintering process, it lacks stiffness and flexibility when producing a filament. Stearic acid, in low quantities, decreases the feedstock viscosity, by reducing the friction between powder and binder. The additives PEC and PA assist this purpose, as they are tougher and more elastic thermoplastics. PEC has very good flexibility, flowability, and a low warpage tendency, while PA adds stiffness. Only that which was required was added to give the filament good extrusion and printing properties. 

When developing a new feedstock composition, there must be a balanced proportion of powder and organic binder. Too much powder will embrittle the composition and make it difficult to process, while too little will open too much space between the particles, thereby leading to unsuitable debinding and sintering results (i.e., defects and porosity) [41,42]. While mixing, after introducing all of the components, there should be a constant mixing force value, which means that homogeneity has been reached. If this mixing value is too high or low, then the mixture may not be extruded into a filament. 

A thermogravimetric (TGA) analysis was conducted to evaluate the best processing temperature of the feedstock while guaranteeing that no polymer degradation happened before debinding and that it would only decompose in different ranges of temperatures.

### 2.2. Processing

Two feedstocks were prepared based on WC-Co and Ti(CN)/WC-Ni/Co powder. The same organic binder and powder loading (50 vol.%) were selected. These feedstocks were mixed in a torque rheometer (Brabender^®^ Plastograph^®^ W 50, Duisburg, Germany) for 60 min at a 30 rpm blade speed and at a 140 °C temperature. After cooling down, they were granulated into small pellets and sieved at 5 mm. 

Filaments were made in a single screw extruder (Brabender GmbH & Co., Duisburg, Germany), at 120 °C; in addition, the screw speed was 4 rpm and the nozzle (which was made of coated hardened steel) diameter was 1.75 mm.

The 3D objects were printed using a 3D printer (Prusa i3 MK3, Prague, Czech Republic), with a 140 °C nozzle temperature (Table 3). The nozzle was made of hardened steel, with a 0.4 or 0.25 mm diameter hole. Other printing parameters were adjusted according to the 3D object shape.

### 2.3. Debinding and Sintering

The polymer and the other organic components were removed only by thermal debinding. This treatment was carried out at a maximum temperature of 600 °C in an H_2_ atmosphere for 8 h. Sintering was performed in two steps between 1300 °C and 1450 °C (maximum), in an argon atmosphere at 30 bar. The debinding and sintering were performed on the same thermal cycle in a SinterHIP furnace.

### 2.4. Characterization Techniques

The characterizations of the powders, feedstocks, and filaments were performed using the following techniques: laser diffraction to measure the particle size (Malvern Mastersizer 3000, Malvern Instruments Ltd., Worcestershire, UK) according to ISO 13320:2020(E) [46]; scanning electron microscopy (SEM), which was used to analyze the structure, morphology, and shape factor (Zeiss MERLIN, 73447 Oberkochen, Germany) (FEI Quanta 400FEG, FEI Europe BV, Eindhoven, The Netherlands); thermogravimetry (TGA), which was utilized to analyze the weight variation kinetics (SDT Q600 V20.9 Build 20, TA Instruments, New Castle, DE, USA); and µCT (X-ray micro-computed tomography), which was used to scan with a Bruker SkyScan 1275 (Bruker, Kontich, Belgium). An acceleration voltage of 100 kV and a beam current of 100 μA were set while using a 1 mm copper filter and the step-and-shoot mode. The pixel size was set to 6 μm, and the random mode was used. In total, 901 projection images were acquired at a 0.4° angular step with a 3-frame average per step while using an exposure time of 245 ms. The micro-CT images were reconstructed with dedicated manufacturer software. Hardness was measured according to ISO 6507-1:2018 with a Struers Duramin (Ballerup, Hovedstaden, Denmark) while using a 9.8 N force for 15s and 10 indentation measurements. The surface roughness and hole mapping were measured using infinite focus measurement (IFM), a non-contact optical 3D measurement device (Alicona Infinite Focus, Raaba, Steiermark, Austria). This device creates a reconstructed 3D image of a surface by taking several high-resolution pictures (2D images covering a certain area) from a low to high focal point. This process has high repeatability and is independent of size, material, geometry, weight, and surface finish. The surface roughness and hole mapping were measured following ISO 21920-3:2021 [47] and ISO 25178-2:2021 [48]. ImageJ software [49] (free source) was used to measure the pixel density of a specific zone in a given area.

## 3. Results and Discussion

### 3.1. Feedstock and Filaments

**Figure 2:**
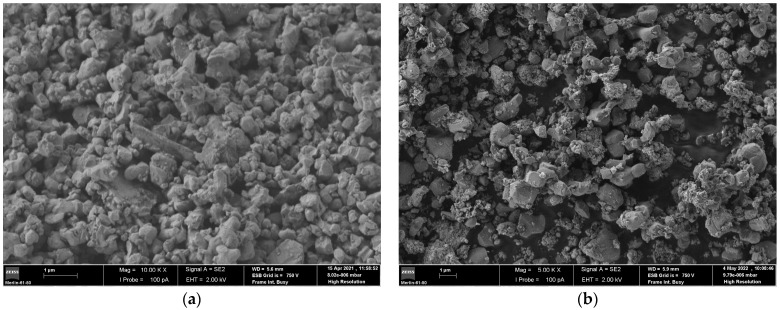
WC-Co (**a**) and Ti(CN)/WC-Ni/Co (**b**) particle shapes (SEM).

The powder used had an irregular shape (Figure 2), particle size below 10 µm, and a narrow particle size distribution. Unlike with other AM processes, a shape factor close to 1 or high flowability are not mandatory for MEX, which means there is a greater freedom to use different sizes and shapes of powders to produce filaments.

**Figure 3:**
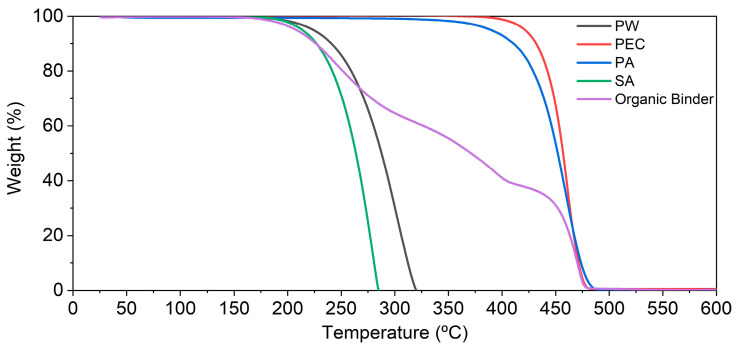
Feedstock and its organic binder degradation behavior (TGA).

A thermogravimetric analysis (Figure 3) of the organic binder and its polymers showed that both PW and SA have the lowest starting degradation temperature at 160 °C. They reached full degradation at 305 °C (SA) and 340 °C (PW). PA started to degrade at 290 ºC and finished at 500 °C, while PEC started at 370 °C and finished at 490 °C. This means that PEC had the narrowest temperature difference. Both PEC and PA leave minimal carbon residue, i.e., below 0.5 wt.%. Since PW and SA transition to a liquid phase by 58 °C (PEC at 84 °C and PA at 120 °C, and where the smallest starting degradation temperature was 160 °C), a processing temperature of between 120 °C and 140 °C was chosen. The organic binder curve, which represents the polymer combination (Table 2), shows a gradual decomposition between 160 °C and 500 °C, with the steepest section occurring in the last 50 °C with the removal of most of the PEC and PA. Complete debinding can be achieved below 600 °C.

**Figure 4:**
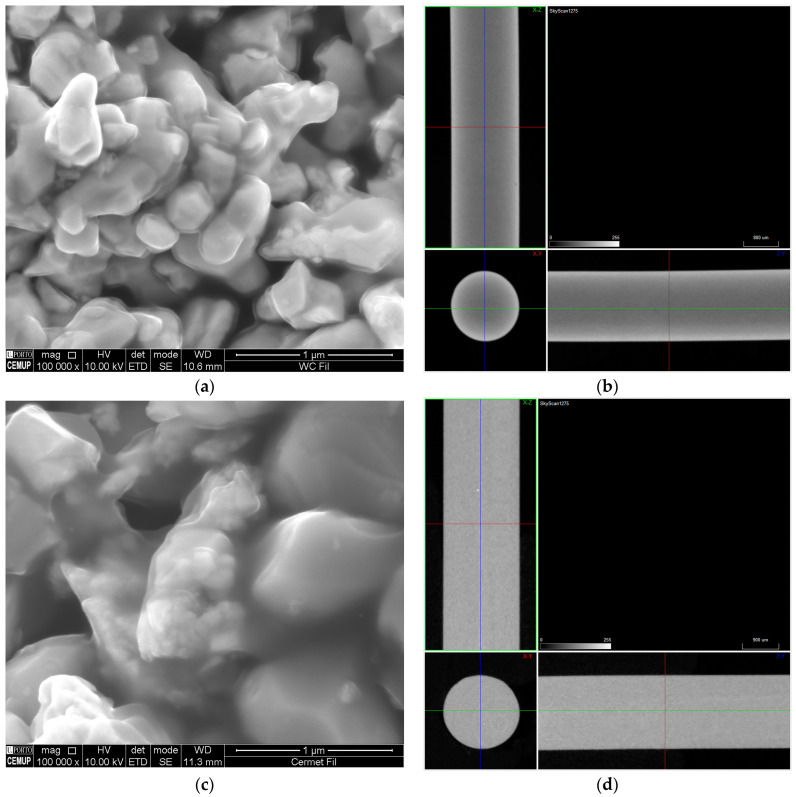
WC-Co (**a**—SEM and **b**—µCT) and cermet (**c**—SEM and **d**—µCT) filaments.

Both feedstocks reached homogeneity before 30 min, with cermet demonstrating a smaller mixing torque than WC-Co (~2 N·m). This may be acceptable due to the fact that cermet powder has half the density of WC-Co, which means there is less weight to mix for the same chamber volume. During filament extrusion, the cermet feedstock had almost half the extrusion pressure of WC-Co. Both filaments were similar (Figure 4), and they demonstrated enough flexibility and stiffness at room temperature to be used in a common 3D printer. Nonetheless, they were still fragile and needed care when handled. If heated to 30–40 °C, they would greatly increase flexibility, and this was due to PW and PEC having low softening temperatures. They were homogeneous in the powder–binder distribution, whereby the organic binder completely coated the powder as a thin layer (Figure 4a,c). They presented a round cross-section, a constant diameter, and a smooth surface (Figure 4b,d).

### 3.2. The 3D Object Surface Roughness

**Figure 5:**
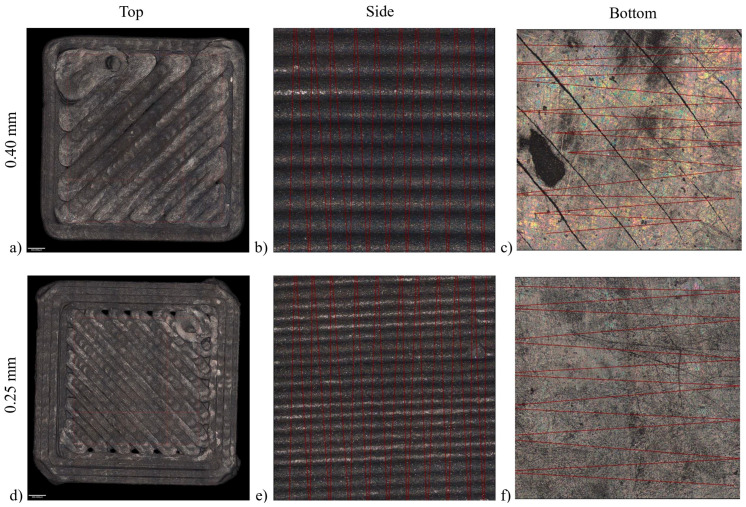
C1 (**a**–**c**) and C2 ((**d**–**f**) IFM).

The MEX 3D objects had a very specific printing pattern that directly influenced the roughness of each of the following main surfaces: top, side, and bottom (Figure 5). The bottom of the green had the lowest values of roughness since the first layer deposited was “squeezed” against the built plate, thus creating a smooth and even surface (Table 4). After sintering, this was the only surface that showed an increase in roughness, which was possibly caused by its contact with the higher roughness of the graphite plates (which molded it during debinding). The side was represented by the stacking of each layer, and it was mostly dependent on the layer height that was chosen for printing. It had the highest roughness of all the three surfaces, with a semi-circle shape for each layer. The top layer had a lower roughness, but a bigger distance between the highest and lowest point. The pattern was adjustable depending on the chosen infill strategy. As expected, the use of a smaller printing nozzle (Figure 5d–f) enabled a smaller surface roughness, since the strand extruded was also smaller. This means that, for more smooth and detailed surfaces, a smaller nozzle should be considered. However, using smaller nozzles increases the printing time considerably, while needing careful machine calibration. To achieve a lower surface roughness, a post-treatment must be considered—either grinding or abrasion.

### 3.3. Hole Size and Orientation

**Figure 6:**
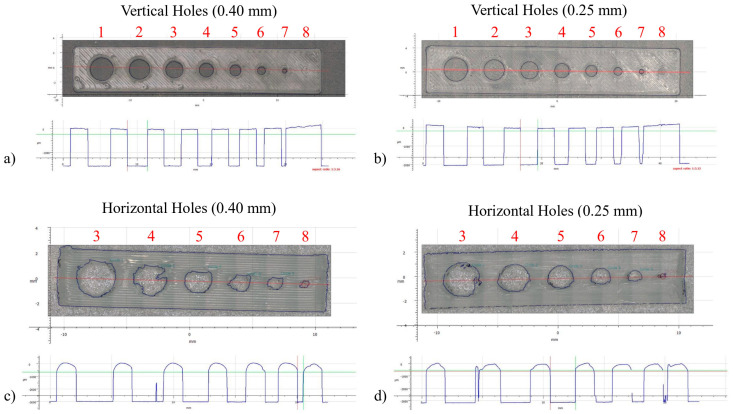
Hole profiles: V1 (**a**); V2 (**b**); H1 (**c**); H2; and (**d**) WC + Co composition (IFM).

Cutting tools and wear-resistant tools need cooling solutions through internal channels. For this reason, vertical and horizontal holes, made by MEX, were analyzed according to the cylindricity and process limitations (Figure 6). The vertical holes were straight and had a good circular shape. By decreasing the hole size, deviations from the nominal value increased, and this was possibly due to the pronounced deformation of the strand when making a circle (Table 5). This is indicated by the values being closer to the nominal value with respect to what the 0.25 mm nozzle achieved compared to the 0.40 mm nozzle. Also, after sintering, there seemed to be a bigger shrinkage for the 0.40 mm vertical holes than the 0.25 mm ones. Hole number 8 was closed for both V1 and V2 (Figure 6), meaning that the smallest vertical hole achieved was number 7 with a 0.79 mm diameter (green). The straightness of the V1 holes was slightly better than that of the V2 ones, even when considering smaller diameter holes. This may be justified by the fact that, while a smaller nozzle can attain greater precision and detail, it can also multiply the number of process defects due to the increased number of layers and strand length. The horizontal holes showed a particularly poor quality. This was expected since overhang structures, without support, are not recommended for AM. However, since the supports could not be removed from the internal channels, they had to be tested accordingly. Even though the diameter did not show a big difference from the nominal value, the standard deviation increased considerably (Table 5). This was clear from the deformed shape, with many irregularities along the hole (Figure 6c,d). The hole circularity was particularly dependable with respect to the number of layers and their size since the built strategy was conducted along the Z axis instead of the XY plane. Increasing the hole size should increase hole precision by decreasing the influence of each layer; however, the possibility of collapse then also increases. Smaller holes tend to be more stable but also show a loss in their circularity; they gain a rectangular shape with rounded side edges. Nonetheless, it was possible to make hole number 8 with a 0.50 mm height (green). When using a smaller nozzle, no significant improvements were observed. For the horizontal holes, new cross-section geometries should be considered, like a water drop shape, since they can be built without overhangs, thus avoiding major deformation.

### 3.4. Defects 

**Figure 7:**
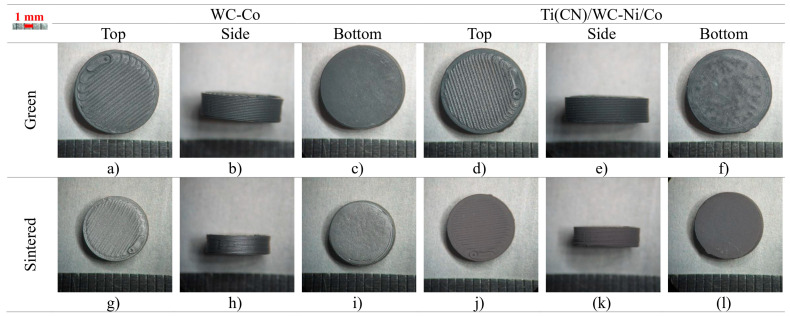
S1, green, and sintered (top, side, and bottom, respectively).

Cylinders with different geometric configurations were analyzed according to their macroscopic defects caused by debinding and sintering. S1 was the first tested shape, and it respected a common powder injection molding (PIM) rule of having a thickness not higher than 3 mm. As expected, there was no deformation in S1 after sintering, for both hardmetal and cermet compositions (Figure 7). The linear shrinkage was similar between the diameter and height, with WC-Co having the higher linear shrinkage of above 21%, while Ti(CN)/WC-Ni/Co was below 20% (Table 6).

**Figure 8:**
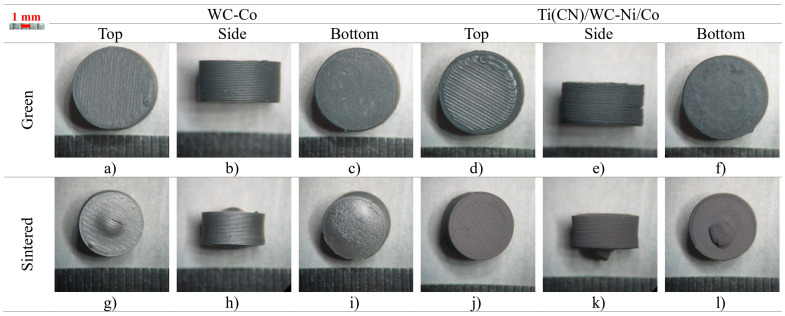
S2, green, and sintered (top, side, and bottom, respectively).

When the height of the cylinder was increased to 5 mm (S2), the point at the bottom (in the center) was more than 3 mm away from the surface, which was not in alignment with the PIM rule of thickness. Both materials showed deformation (Figure 8). The WC-Co had a bubble formation on the top surface and a pronounced curvature on the sides. The organic binder gases, which were trapped inside, deformed the cylinder before being released. In the Ti(CN)/WC-Ni/Co, the material was ejected from the bottom surface while also presenting a curvature on the sides, which was possibly due to the lack of material inside. Due to deformation, the linear shrinkage was irregular between the diameter and height, which thus produced higher standard deviation values (Table 7).

**Figure 9:**
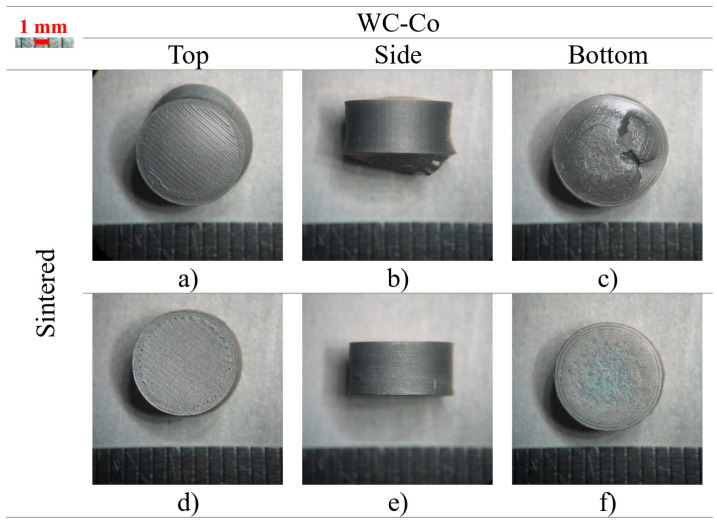
S3 sintered with defects (**a**–**c**) and no defects (**d**–**f**) (top, side, and bottom, respectively).

S3 was found to be like S2, but it was also printed with a smaller nozzle diameter of 0.25 mm. Although certain samples showed the same type of deformations found on S2 (Figure 9a–c), the others did not show anything major (Figure 9d–f). All S2 samples showed deformation, meaning that using a smaller nozzle may have helped to release some of the gas trapped inside. It could be speculated that a smaller nozzle can lead to smaller porosity but also a higher quantity at the same time. Also, since each strand was thinner, the surface area exposed inside the 3D object, was probablyhigher than the one from a thicker strand. The linear shrinkage was found to be similar between the diameter and height (21 and 22%) (Table 8), and it was also in accordance with the values for S1 (WC-Co).

**Figure 10:**
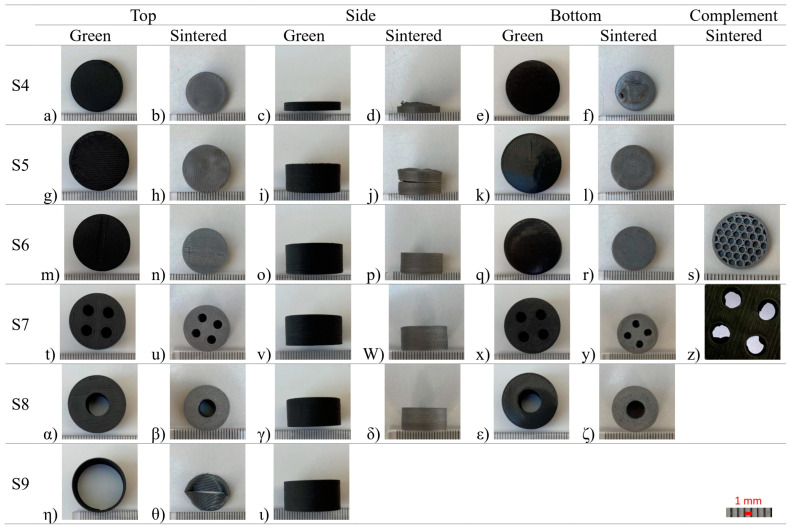
S4, S5, S6, S7, S8, S9, green, and sintered (top, side, bottom, and complement, respectively).

The S4 cylinder, with its 3 mm height, maintained the PIM rule of thickness; however, it had an increase in diameter from 10 to 20 mm, and there was a bloating effect at the bottom, causing deformation (Figure 10d,f). This showed that even thin objects may be subjected to deformation if the total mass is increased. As expected in S5, by greatly increasing the volume of a fully dense 3D object, further deformation was detected on the side with a crack propagation at the center, causing delamination (Figure 10j). This opening was caused by the extreme pressure accumulated inside, which escaped from the weakest point of the 3D object, i.e., the inter-layer connection, with each layer only being stacked on top of the other. To demonstrate one of the capabilities of MEX in relation to the conventional pressing and sintering process, S6 was built with the same volume as S5 but with a 30% infill honeycomb pattern (Figure 10s). S6 did not show any external deformation and kept its shape. This is possible because the open structure inside created artificial “chimneys” for the organic gases to flow and be released easily from the 3D object. The linear shrinkage for the diameter was 21%; while for the height, it was 25% (Table 9). It should be noted that this study only considered a debinding and sintering point of view, not a mechanical perspective. S7 and S8 included vertical holes that served two functions: helping with debinding and sintering, and being considered as cooling channels for cutting tools (Figure 10t) α)). Neither showed external deformation, and the linear shrinkage for S8 was equal to that for S1 and S3 (i.e., no defects), while S7 had small shrinkage values. However, it should be noted that S7 did show deformation along the four smaller diameter holes (Figure 10z)). Some of the holes became oval, with a bloating effect on the walls that could have led to channel obstruction. S8, with a single bigger single hole, had no deformation. Nonetheless, the linear shrinkage for the holes was higher for both at 25%. Even though S7 and S8 had similar cross-section areas for the holes, one single hole of 8 mm was more effective at assisting in debinding than four holes with 4 mm diameters. Finally, S9 served as a thin wall test. The stresses created during sintering were enough to deform the 3D object, leading to its collapse. This was a demonstration that too-thin walls, without supports, are prone to warping.

### 3.5. Microstructure

**Figure 11:**
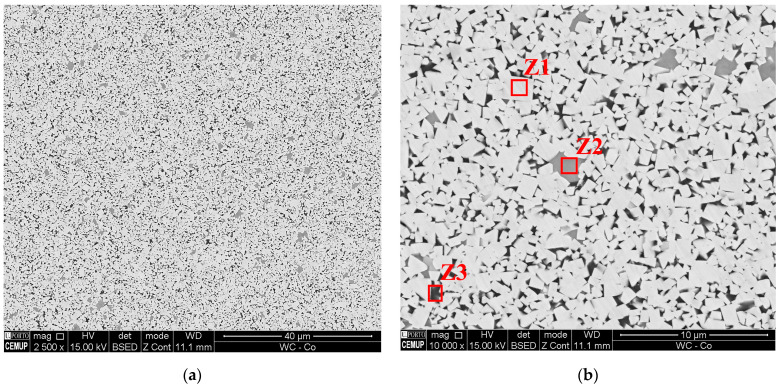
The WC-Co microstructure made by MEX: 2500× (**a**) and 10,000× (**b**).

**Figure 12:**
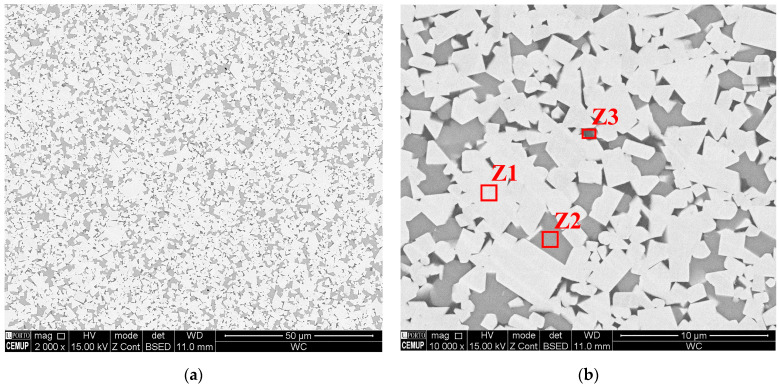
The WC-Co microstructure made by the pressing and sintering method: 2500× (**a**) and 10,000× (**b**).

After the different stages of MEX, the WC-Co 3D objects had a homogeneous microstructure with no evidence of porosity, graphite, or the η phase (Figure 11). Three main zones were identified: tungsten carbide (Z1), grain growth inhibitors (Ti, Cr, Ta, and Nb) (Z2), and cobalt (Z3) (Figure 11b). If compared with the pressing and sintering microstructures (Figure 12), the size of WC (Z1) and the content of the grain growth inhibitors (Z2) were smaller. The hardness was similar between the MEX and the conventionally pressed and sintered 3D objects, using the same powder (Table 10). The microstructure corresponded to a submicron WC grade (~8 µm).

**Figure 13:**
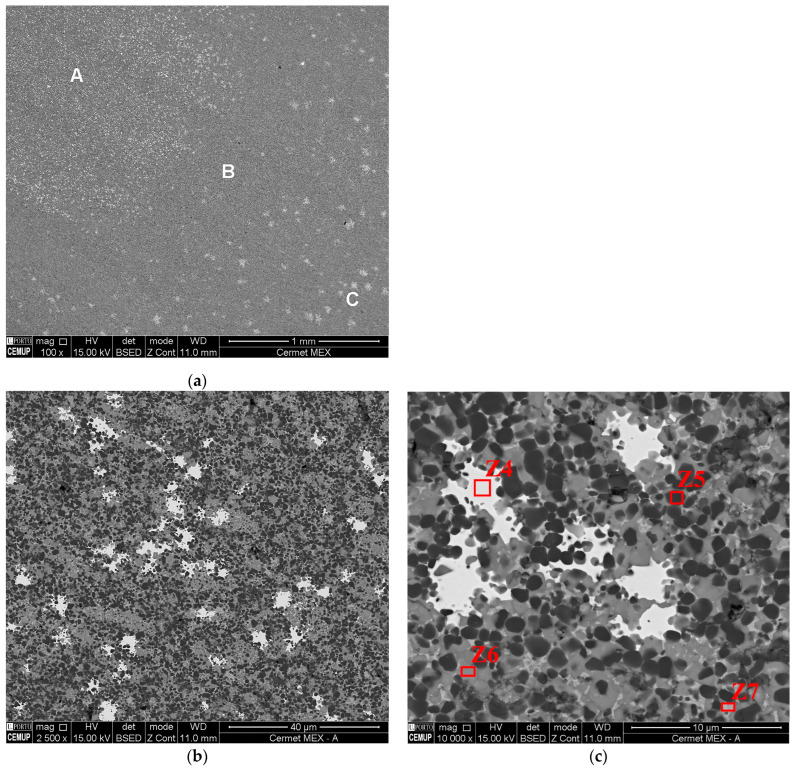

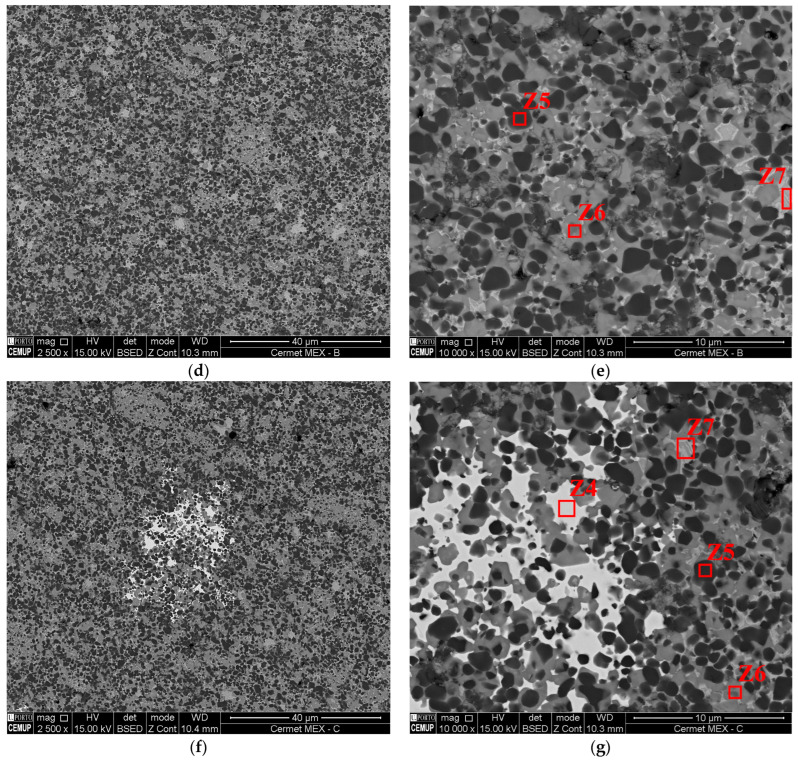
Cermet microstructure made by MEX (**a**); Area A (**b**,**c**); Area B (**d**,**e**); and Area C (**f**,**g**).

Unlike WC-Co, the cermet 3D objects that were manufactured by MEX had a heterogeneous microstructure. Three different areas were identified (A, B, and C) (Figure 13a). The main difference between them was the distribution and number of tungsten-enriched zones (Z4) (Figure 13c). Area A was enriched in W with very small, dispersed zones (Figure 13b,c). Area B was homogeneous without significant tungsten-enriched zones (Figure 13d,e). Moreover, Area C was constituted by dispersed large zones based on tungsten (Figure 13f,g). 

Nevertheless, all the main heterogeneous areas were mostly composed of Ti (Z5), Ti with other elements (Z6), low Ti content (Z7), and tungsten-enriched zones (Z4). As the amount of Ti decreased, the W increased. Co and Ni (metallic binders) were mainly present in the low-Ti-content zones (Z7). Mo and Nb had similar quantities in Z4, Z6, and Z7. 

The hardness of the 3D objects produced via MEX was lower than that of objects obtained via the conventional pressing and sintering process (Table 10). The lakes of the binders were observed as typical in the conventional-processed microstructures, where the pore zones formed due to insufficient pressure during the uniaxial pressing stage, were filled with the binder during the sintering stage.

**Figure 14:**
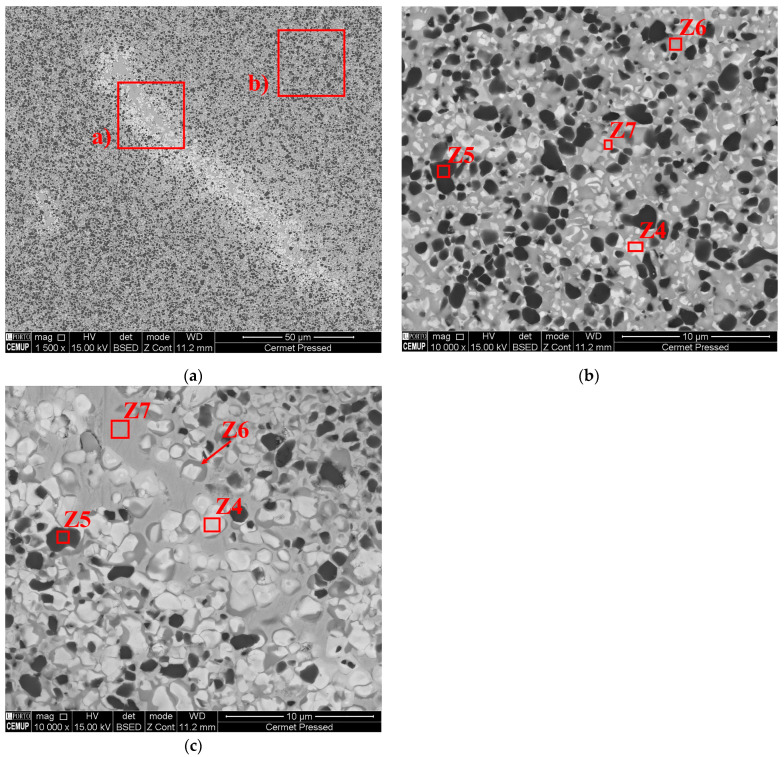
The cermet microstructure after the pressing and sintering process (**a**); those that were constituted predominantly by homogeneous zones (**b**); and those that possessed some heterogeneous zones (**c**).

Cermets processed by the pressing and sintering process have a microstructure that is evidently more homogeneous than those that result from MEX (Figure 14b)). The microstructures present limited heterogeneities, and they were enriched in Co and Ni (metallic binders) (Figure 14a,c). Compared with MEX, the tungsten-enriched zones (Z4) (Figure 14b,c) had a lower presence of W, and they were more evenly distributed between Z6 and Z7. The lack of homogeneity, after MEX, could be a result of the different properties and characteristics of the powder particles that constitute the cermet. These can promote, during filament processing, a heterogeneous distribution of different powder materials. The detailed analyses of the filaments after sintering could lead to a better understanding of the observed MEX-processed 3D objects.

**Figure 15:**
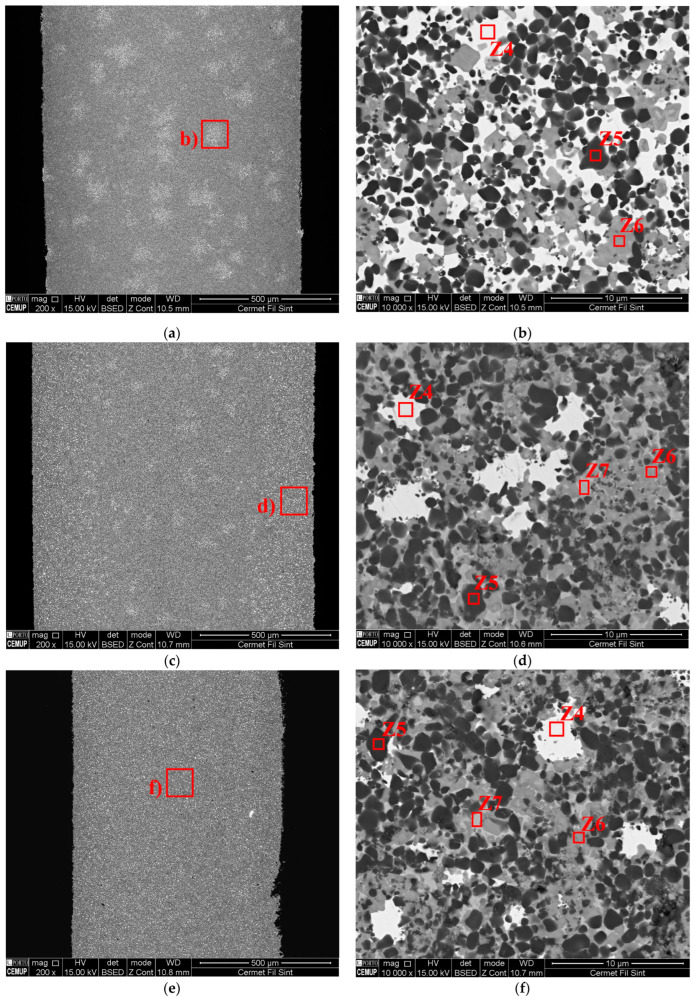
The cermet microstructure in three sections of the sintered filament: Area C-like pattern (**a**,**b**); Area C-and-A-like pattern (**c**,**d**); and Area A-like pattern (**e**,**f**).

The presence of W-rich zones (Z4) was also found on the sintered filaments within the three main areas identified previously (Figure 13a). In one section, the Area C pattern was predominant (Figure 15a,b), while, in another, Area C was found in the middle and Area A in the periphery (Figure 15c,d). Other filament sections showed only an Area A pattern (Figure 15e,f). This behavior could have an important role in the heterogeneity of MEX-processed 3D objects.

## 4. Conclusions

This study focused on the differences, in terms of the macro-to-microscopic defects, between WC-Co and cermet behaviors during MEX processing. 

High-quality filaments for MEX were successfully produced from WC-Co. However, the filaments in the cermet feedstock (Ti(CN)/WC-Ni/Co) had, in similar extrusion conditions, microstructural heterogeneities with different chemical compositions, and these were present from the center to the periphery. It was demonstrated that this behavior had an important role in the quality of the tools.

WC-Co 3D objects have the same microstructure and hardness as the ones made by the pressing and sintering process. Nevertheless, Ti(CN)/WC-Ni/Co tools, due to their heterogeneous microstructure, saw a significant decrease in their hardness compared with the same powders when processed by conventional technology. 

As the main conclusion, it must be highlighted that this research contributed to the establishment of the geometric boundaries that are required to successfully produce a complex system of cooling channels inside a WC-based tool when using MEX technology (with whatever geometry is needed). Unfortunately, the current knowledge is not yet sufficient for these findings to be applied to Ti(CN)/WC-Ni/Co tools, and this is essentially due to the heterogeneity of their microstructure.

## Figures and Tables

**Table 1:** Powder characteristics.

Powder	Specific Weight (Kg/m^3^)	D10 (µm)	D50 (µm)	D90 (µm)	Metal Binder (wt.%)
WC-Co	14,250 ± 100	0.82	1.74	4.63	10
Ti(CN)/WC-Ni/Co	6600 ± 50	1.15	2.13	3.81	15

**Table 2:** Feedstock composition.

Powder [vol.%]	Organic Binder [vol.%]		
50	50		
	PW	PEC and PA	SA
	40	50	10

**Table 3:** The 3D objects created with MEX.

	Shape	Dimension [mm]	Nozzle Diameter [mm]	Infill [%]	Hole Diameter [mm]	Material
C1	Cube [L W H]	6.00 × 6.00 × 6.00	0.40	100	-	WC + Co
C2	Cube [L W H]	6.00 × 6.00 × 6.00	0.25	100	-	WC + Co
V1	Rect. ^1^ [L W H]	43.00 × 8.00 × 2.00	0.40	100	4.00 to 0.50 (8 holes)	WC + Co
V2	Rect. ^1^ [L W H]	43.00 × 8.00 × 2.00	0.25	100	4.00 to 0.50 (8 holes)	WC + Co
H3	Rect. ^1^ [L W H]	21.70 × 3.00 × 4.60	0.40	100	3.00 to 0.50 (6 holes)	WC + Co
H4	Rect. ^1^ [L W H]	21.70 × 3.00 × 4.60	0.25	100	3.00 to 0.50 (6 holes)	WC + Co
S1	Cyl. ^2^ [D H]	10.00 × 3.00	0.40	100	-	WC and Ti(CN)/WC-Ni/Co
S2	Cyl. ^2^ [D H]	10.00 × 5.00	0.40	100	-	WC and Ti(CN)/WC-Ni/Co
S3	Cyl. ^2^ [D H]	10.00 × 5.00	0.25	100	-	WC + Co
S4	Cyl. ^2^ [D H]	20.00 × 10.00	0.40	100	-	WC + Co
S5	Cyl. ^2^ [D H]	20.00 × 10.00	0.40	100	-	WC + Co
S6	Cyl. ^2^ [D H]	20.00 × 10.00	0.40	30 (honeycomb)	-	WC + Co
S7	Cyl. ^2^ [D H]	20.00 × 10.00	0.40	100	4.00 (4 holes)	WC + Co
S8	Cyl. ^2^ [D H]	20.00 × 10.00	0.40	100	8.00 (1 hole)	WC + Co
S9	Cyl. ^2^ [D H]	20.00 × 10.00	0.40	0	19.20 (thin wall)	WC + Co

^1^ Rectangular cuboid. ^2^ Cylinder. L—length; W—width; H—height; D—diameter.

**Table 4:** Surface roughness of C1 and C2.

Cube		Average Height of Selected Area—Sa (µm)			Maximum Height of Selected Area—Sz (µm)		
		Top	Side	Bottom	Top	Side	Bottom
Green	C1 (0.40)	5.71	19.69	0.90	171.64	146.19	34.58
	C2 (0.25)	3.69	9.49	0.70	158.18	107.36	9.42
Sintered	C1 (0.40)	4.42	14.86	2.37	152.15	106.78	36.17
	C2 (0.25)	2.61	7.77	6.13	104.84	65.17	130.88

**Table 5:** Hole diameter measurements.

			Circle Diameter (mm)															
			#1		#2		#3		#4		#5		#6		#7		#8	
Nominal (Green)			4.00		3.50		3.00		2.50		2.00		1.50		1.00		0.50	
Green	Vertical Holes	0.40	3.96	±0.02	3.49	±0.03	2.96	±0.03	2.41	±0.02	1.90	±0.02	1.35	±0.01	0.79	±0.01	blocked	blocked
		0.25	4.01	±0.02	3.49	±0.02	2.95	±0.02	2.45	±0.02	1.94	±0.02	1.42	±0.03	0.85	±0.03	blocked	blocked
	Horizontal Holes	0.40					3.04	±0.17	2.60	±0.33	2.12	±0.24	1.57	±0.17	1.07	±0.15	0.50	±0.13
		0.25					2.84	±0.23	2.49	±0.17	2.09	±0.99	1.54	±0.14	1.11	±0.14	0.41	±0.09
Sintered	Vertical Holes	0.40	3.08	±0.02	2.75	±0.03	2.34	±0.04	1.92	±0.03	1.49	±0.02	1.07	±0.02	0.62	±0.02	blocked	blocked
		0.25	3.13	±0.02	2.80	±0.04	2.37	±0.04	1.98	±0.03	1.57	±0.03	1.15	±0.04	0.67	±0.04	blocked	blocked
	Horizontal Holes	0.40					2.40	±0.14	1.70	±0.17	1.64	±0.21	1.19	±0.14	0.82	±0.10	0.40	±0.12
		0.25					2.01	±0.16	1.96	±0.07	1.62	±0.08	1.22	±0.05	0.80	±0.05	0.33	±0.03

**Table 6:** S1 geometric measurements.

[mm]	WC-Co		Ti(CN)/WC-Ni/Co	
	Diameter	Height	Diameter	Height
Nominal	10.00	3.00	10.00	3.00
Green	10.00 ± 0.05	3.02 ± 0.01	10.06 ± 0.04	3.06 ± 0.01
Sintered	7.83 ± 0.10	2.38 ± 0.03	8.08 ± 0.03	2.48 ± 0.04
Shrinkage	21.7%	21.2%	19.7%	19.0%

**Table 7:** S2 geometric measurements.

[mm]	WC-Co		Ti(CN)/WC-Ni/Co	
	Diameter	Height	Diameter	Height
Nominal	10.00	5.00	10.00	5.00
Green	9.95 ± 0.02	4.99 ± 0.11	10.17 ± 0.01	5.13 ± 0.04
Sintered	7.62 ± 0.04 (+0.37 max.)	3.95 ± 0.10 (+1.48 max.)	8.13 ± 0.10 (+0.10 max.)	4.27 ± 0.06 (+1.57 max.)
Shrinkage	23.4%	20.8%	20.1%	16.8%

**Table 8:** S3 geometric measurements.

[mm]	Defect		No Defect	
	Diameter	Height	Diameter	Height
Nominal	10.00	5.00	10.00	5.00
Green	9.91 ± 0.03	4.98 ± 0.02	9.91 ± 0.03	4.98 ± 0.02
Sintered	7.61 ± 0.10 (+0.62 max.)	3.88 ± 0.05 (+1.8 max.)	7.70 ± 0.05	3.92 ± 0.02
Shrinkage	23.2%	22.1%	22.3%	21.3%

**Table 9:** S4, S5, S6, S7, S8, and S9 geometric measurements.

[mm]		Diameter	Height	Hole Diameter
S4	Nominal	20.00	3.00	-
	Green	19.96	3.06	-
	Sintered	15.60	2.33 (4.37 max.)	-
	Shrinkage	21.8%	23.9%	-
S5	Nominal	20.00	10.00	-
	Green	20.06	10.15	-
	Sintered	15.58 (16.24 max.)	7.99 (9.71 max.)	-
	Shrinkage	22.3%	21.3%	-
S6	Nominal	20.00	10.00	-
	Green	19.85	10.03	-
	Sintered	15.68	7.55	-
	Shrinkage	21.0%	24.7%	-
S7	Nominal	20.00	10.00	4.00/4.00/4.00/4.00
	Green	19.96	10.07	3.87/3.81/3.82/3.84
	Sintered	16.02	8.26	2.89/2.93/2.91/2.92
	Shrinkage	19.7%	18.0%	25.0%
S8	Nominal	20.00	10.00	8.00
	Green	19.94	10.02	7.8
	Sintered	15.6	7.9	5.8
	Shrinkage	21.8%	21.2%	25.6%
S9	Nominal	20.00	10.00	19.20
	Green	19.86	9.96	19.04
	Sintered	Colapse		
	Shrinkage			

**Table 10:** Hardness measurements.

	WC-Co		Ti(CN)/WC-Ni/Co	
	Pressing-and-Sintering	MEX	Pressing-and-Sintering	MEX
Hardness [HV1/15]	1702 ± 28	1649 ± 24	1872 ± 23	1265 ± 60

## Data Availability

Data sharing is not applicable to this article.

## Nomenclature

MEX	Material extrusion
AM	Additive manufacturing
PBF	Powder bed fusion
BJT	Binder jetting
VPP	Vat photopolymerization
SLS	Selective laser sintering
SEM	Scanning electron microscopy
TGA	Thermogravimetric
TPE	Thermoplastic elastomer
PP	Polypropylene
RTP	Ready-to-press
PW	Paraffin wax
PEC	Poly(propylene-ethylene) copolymer
PA	Polyamide
SA	Stearic acid
PIM	Powder injection moulding
IFM	Infinite focus measurement
